# Dynamic R2' Imaging can Be a Biomarker for Diagnosing and Staging Early Acute Kidney Injury in Animals

**DOI:** 10.3389/fmed.2021.775042

**Published:** 2021-12-24

**Authors:** Bihui Zhang, Ziping Yao, Weizheng Gao, Chengyan Wang, Hanjing Kong, Jue Zhang, Min Yang

**Affiliations:** ^1^Department of Interventional Radiology and Vascular Surgery, Peking University First Hospital, Beijing, China; ^2^Academy for advanced interdisciplinary studies, Peking University, Beijing, China; ^3^Human Phenome Institute, Fudan University, Shanghai, China; ^4^Beijing United Imaging Research Institute of Intelligent Imaging, UIH Group, Beijing, China

**Keywords:** magnetic resonance imaging, acute kidney injury, hemodynamic response imaging, diagnosis, biomarker (BM)

## Abstract

**Background:** Early diagnosis of acute kidney injury (AKI) is essential in clinical settings. None of the current biomarkers are widely applied. The combination of pulse-shifting multi-echo asymmetric spin-echo sequence (psMASE) and a modified hemodynamic response imaging (HRI) technique is promising. The purpose of this study was to evaluate the feasibility of psMASE combined with HRI in detecting early ischemic AKI in animal models of different severities.

**Methods:** Twenty rabbits were divided into four groups (mild, moderate, and severe AKI and control groups). Transarterial embolization with different doses of microspheres was performed to establish AKI animal models of different severities. The 3T psMASE and HRI scans of kidneys were conducted. The R2^*^, R2, and R2' during room air and gas stimulation were acquired and the difference of R2' (dR2') was evaluated in different AKI groups.

**Results:** The values were not different in R2^*^ and R2 during room air and in R2^*^ and R2, and R2' during gas stimulation. The value of R2' was significantly different during room air (*P* = 0.014), but the difference was only found between control and moderate/severe AKI groups (*P* = 0.032 and 0.022). The values of dR2' were different among groups (*P* < 0.0001) and differences between every two groups except comparison of moderate and severe AKI groups were significant (*P* < 0.01).

**Conclusion:** The dR2' imaging acquired by a combination of renal psMASE and HRI technique can serve as a potential quantitative biomarker for early detection and staging of AKI.

## Introduction

Acute kidney injury (AKI) is a multifaceted syndrome associated with increased morbidity and mortality that occurs in ~20% of hospitalized patients (1, 2). It is characterized by a sudden decrease in glomerular filtration rate followed by an increase in serum creatinine concentration or oliguria (1). Treatments for established AKI have been disappointing. Thus early detection or recognition is crucial in research and clinical settings to prevent progression to more severe stages (3). Although serum creatinine is one of the standard diagnostic tools for AKI, it is a delayed and insensitive biomarker of changes in renal function. Moreover, it does not differentiate between functional and structural kidney damages (4). Although several novel biomarkers, including neutrophil gelatinase-associated lipocalin (NGAL), kidney injury molecule-1 (KIM-1), interleukin18 (IL-18), have been introduced to identify early AKI before creatinine rise, the utility is confined to research and clinical applicability is impeded (4–6). Biomarkers that can provide real-time, patient-specific, and pathophysiology-related information of AKI are in demand (6).

Magnetic resonance imaging (MRI) has been identified as a promising modality for identifying early AKI (7–9). Blood oxygenation level-dependent (BOLD) technique, by which deoxyhemoglobin is used as an endogenous contrast agent to probe tissue oxygenation, has been applied to evaluate renal oxygenation status (8, 10). T2^*^ weighted images of BOLD can provide quantitative information about the changes of tissue deoxyhemoglobin. R2^*^ (1/T2^*^) is comprised of two components: R2' and R2. R2' reflects tissue hemoglobin magnetic sensitivity and oxygenation, while R2 is sensitive to the change of tissue water content, interfering with the evaluation of oxygenation (11). Therefore, the acquisition of R2' is needed to measure oxygenation quantitatively. It can be achieved by measuring R2, and R2^*^ simultaneously and dynamically with the help of a pulse-shifting multi-echo asymmetric spin-echo sequence (psMASE) with a moving estimation strategy (12). Moreover, hemodynamic response imaging (HRI), a combination of MRI and respiratory challenges, helps to enhance the sensitivity of renal oxygenation evaluation and AKI detection in animal models (13). A combination of psMASE and HRI can be used to acquire changes of R2' between gas stimulation and room air (difference of R2', dR2'). In this way, it seems to be promising for theoretically detecting and staging AKI in the early phase.

In this study, we combined psMASE sequence with a modified HRI technique to acquire dR2' and evaluated the feasibility of this new method in detecting and staging early ischemic AKI in animal models of different severities.

## Materials and Methods

### Animal Study

Animal experiments were conducted following the guidelines and approval of the institutional animal protection committee (J201740). The model establishement methods were similar as previously reported (8). Twenty New Zealand white rabbits (male, weight range: 2.5–3.5 kg) were randomly divided into mild, moderate, and severe AKI groups and control groups, with five rabbits in each group. Transarterial embolization with different doses of microspheres was performed to establish AKI animal models. The embolization procedure was performed in the angiographic unit (Innova 4100, GE Healthcare, Waukesha, WI). During the procedure, animals were positioned supine on the angiography table and anesthesia was induced by an intravenous injection of pentobarbital sodium. The right femoral artery was dissected after anesthesia and a 4F sheath was inserted after the puncture. A 4F (French) Cobra catheter (Terumo, Beijing, China) was positioned at the ostium of the right renal artery under fluoroscopic guidance. Through the catheter, microspheres (Hepasphere, Merit, Rockland, MD) of 100–150 μm in diameter were injected into the right kidney at a low speed to avoid backflow. Angiography (flow velocity = 1.5 ml/s, flow time = 3 sec) was performed before and after the embolization in all rabbits. Two milliliters of suspension containing 20,000, 40,000, and 60,000 microspheres were administered in the mild, moderate, and severe AKI groups. In the control group, 2 ml saline was administrated. The number of microspheres was counted in the suspension under a microscope of 400 times. The procedure was performed by an interventional radiologist with 6 years of experience. Blood samples were collected from all animals before, 1, 7, 14, and 28 days after the procedure. Serum creatinine levels were determined from the blood samples by using an automatic biochemistry analyzer (Reflotron system, Roche Diagnostics, Basel, Switzerland).

### MRI Scan Protocol

**Figure 1:**
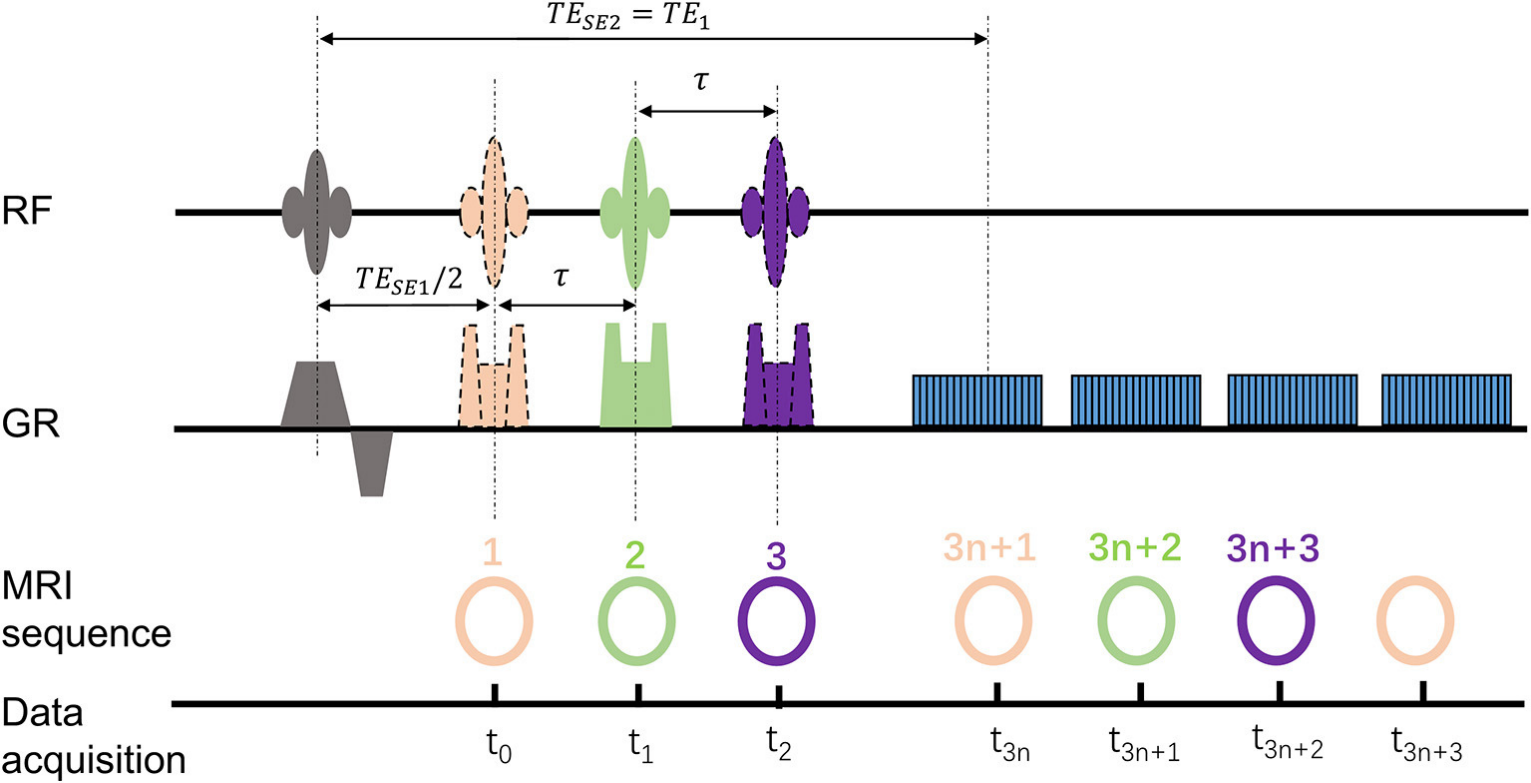
Diagram of the psMASE sequence.

Within 1 h after the embolization procedure, the rabbits were transferred to an MRI table for scanning. The MRI experiments were carried out on a 3.0 Tesla MR scanner (Achieva, Philips Medical Systems, Best, Netherlands). All the rabbits were subjected to the gas challenges by alternating room air and carbogen mixture (5% of CO_2_ and 95% of O_2_) every 5 mins for a total of 20 mins. The psMASE scan was performed during room air and carbogen consecutively. The diagram of the psMASE sequence is displayed in Figure 1. A four-echo asymmetric spin-echo (ASE) EPI sequence with periodic shifted 180° pulse was implemented dynamically. The detailed renal psMASE imaging parameters were: FOV = 150 × 150 mm, matrix size = 70 × 70, TR = 2,000 ms, TE1/TE2/TE3/TE4 = 60/80/100 /120 ms, echo space = 20 ms, τ = −10/0/10 ms, slice thickness = 5 mm, SENSE factor = 2. For the next 4 weeks, coronal and axial T2 weighted imaging scans were performed every week. The coronal scan covered the entire volume of both kidneys and the axial scan covered bilateral kidneys simultaneously. The scan parameters were as follows: FOV = 140 × 120 mm^2^; matrix size = 140 × 140; TR = 3,000 ms; TE = 80 ms; echo space = 20 ms; slice thickness = 5 mm; SENSE factor = 2.

### Quantitative Analysis

The outlining of ROI (region of interest) was performed manually by radiologists with more than 5 years of experience. For AKI groups, regions of 5 × 5 pixels^2^ with lesions as the center on the T2w images were considered ROIs. For the control group, regions of the same area were selected. The quantitative R2' maps were generated as reported previously (12), and the changes of R2' (dR2') during room air and carbogen were calculated. Two-dimensional affine registration was performed to mitigate respiratory motion artifacts. All the processing procedures were programed with MATLAB (MathWorks Inc., Natick, MA, USA).

### Renal Histological Evaluation

The animals were executed one month after the embolization procedure and the kidneys were perfused with saline through the left ventricle until the renal cortex was cleared entirely of blood. Tissue samples were then taken and embedded in paraffin. The paraffin sections were then stained with hematoxylin and eosin (H&E) for histological examinations. The histological analysis was performed by a pathologist with more than 12 years of experience.

### Statistical Analysis

One-way analysis of variance (ANOVA) with Tukey test was used for data analysis between groups and a paired *t*-test was used for tests within the same group. Statistical analyses were performed using SPSS 22.0 software (SPSS, Inc., Chicago, IL, USA) and Graphpad Prism 9.0 (GraphPad Software, San Diego, CA, USA). The difference was significant with *p*-value <0.05.

## Results

**Figure 2:**
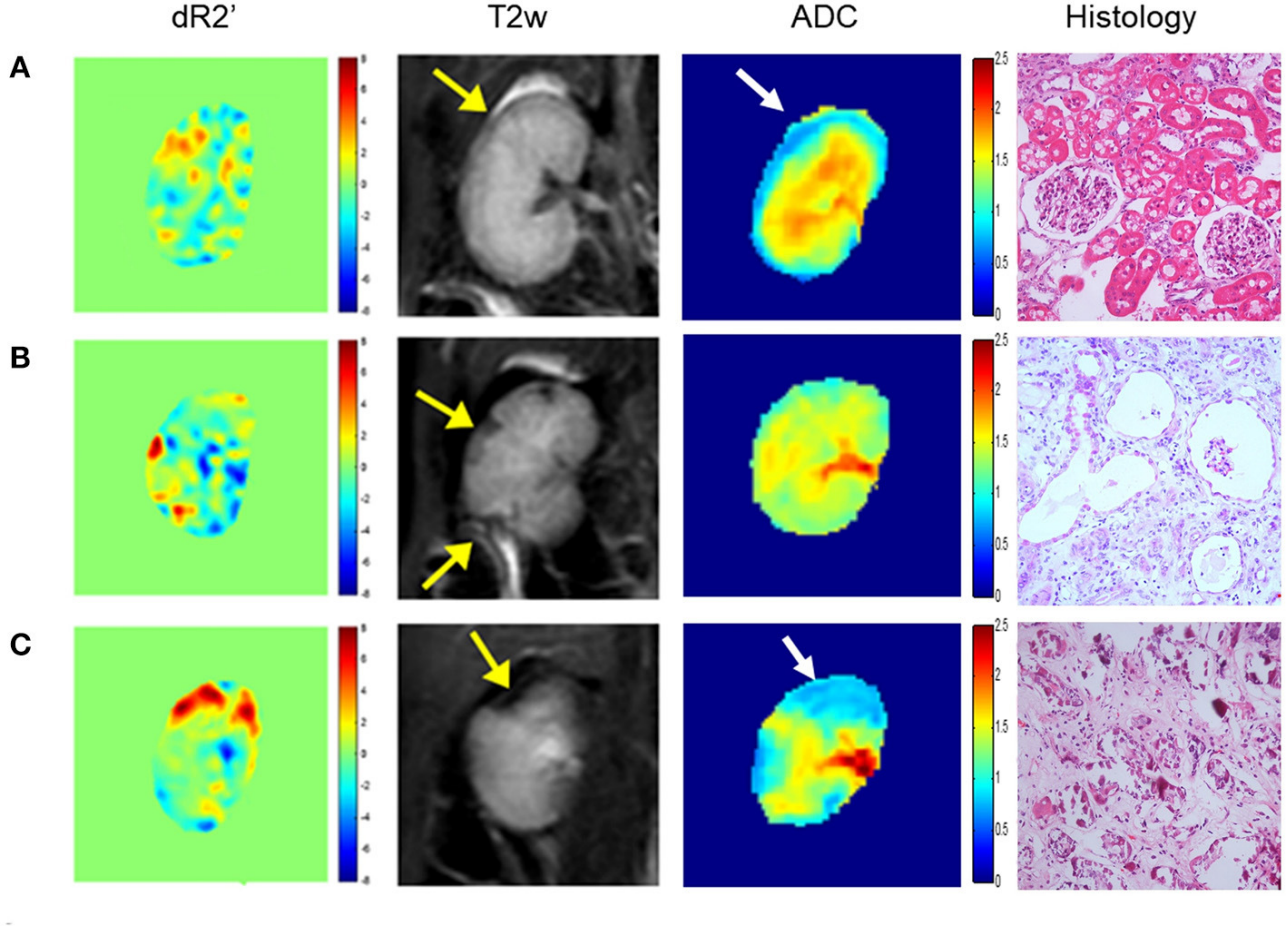
Representative dR2' maps, T2w maps, ADC maps, and histological results of mild **(A)**, moderate **(B)** and severe **(C)** AKI groups. The dR2' and ADC maps are acquired within 1 h after the embolization procedure. The T2w maps and histological results are acquired one month after the procedure. The yellow and white arrows indicate the most affected areas in T2w and ADC images. In the histological images, mild vacuole degeneration occurs in a few renal tubular epithelial cells in the mild case. Atrophy, ischemia and sclerosis of glomerulus and cellular debris are observed in the moderate case. Necrosis and abscission of the renal tubular epithelial cells is observed, and the basement membrane is exposed in the severe case. Atrophy, ischemia and sclerosis of glomerulus can also be observed.

**Figure 3:**
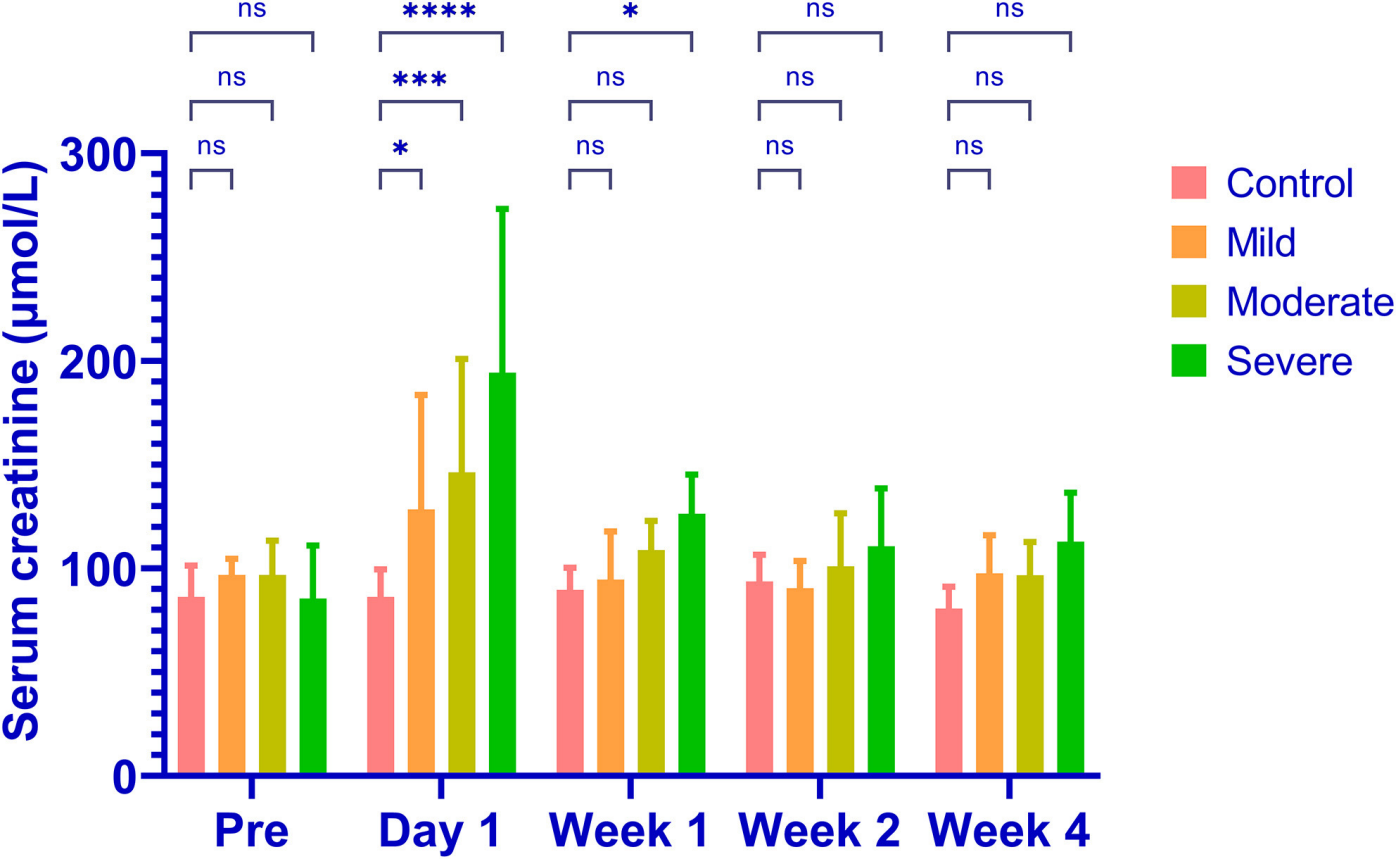
The levels of serum creatinine in different groups. The serum creatinine increases in all AKI groups and the levels of increase are aligned with the severities (control 86.20 ± 10.71μmol/L, mild 128.4 ± 44.44 μmol/L, moderate 146.2 ± 44.02 μmol/L, severe 194.2 ± 63.59 μmol/L). The serum creatinine returns to baseline levels in all groups after 4 weeks. ^*^P < 0.05; ^***^P < 0.001; ^****^P < 0.0001; ns, not significant.

Renal embolization and MRI scans were successfully performed in all rabbits (N = 20). Representative images of lesions in mild (a), moderate (b) and severe (c) AKI groups are shown in Figure 2. The calculated renal dR2' maps within 1 h after the embolization procedure, T2w images and histological results at 1 month of each group are displayed. The histological findings reflected the severities of AKI. The serum creatinine levels elevated after embolization and decreased to baseline levels after 4 weeks in AKI groups (Figure 3). The levels of serum creatinine at Day 1 were aligned with the severities of AKI (control 86.20 ± 10.71 μmol/L, mild 128.4 ± 44.44 μmol/L, moderate 146.2 ± 44.02 μmol/L, severe 194.2 ± 63.59 μmol/L).

**Figure 4:**
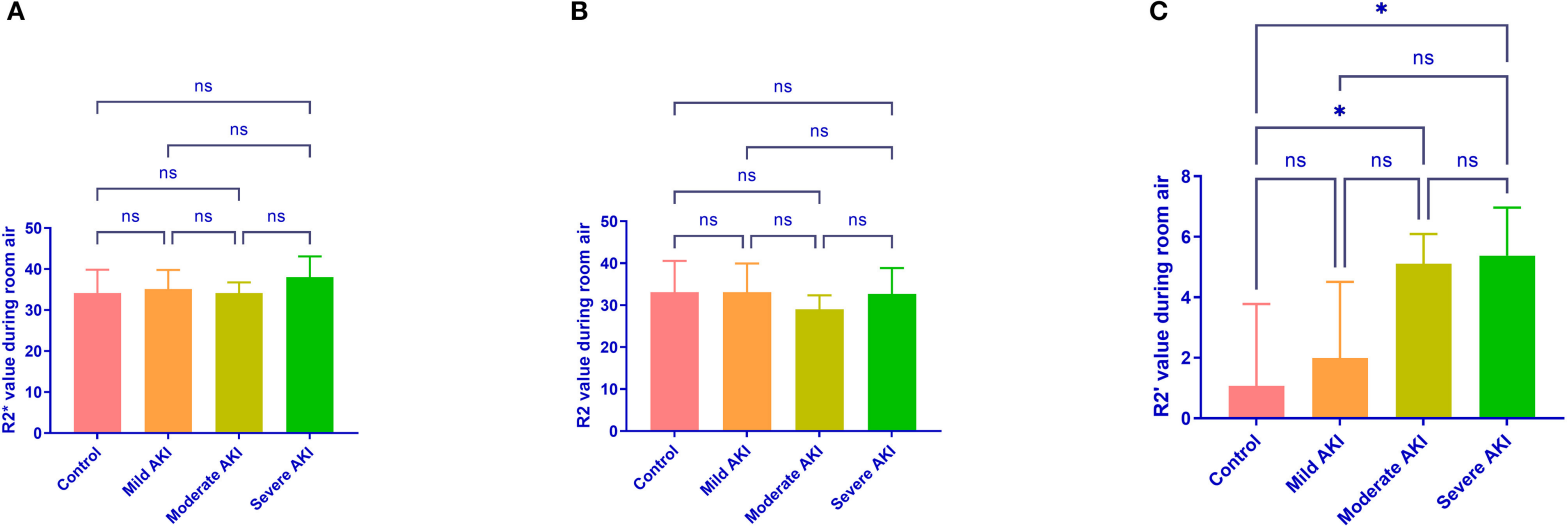
R2^*^, R2, and R2' values during room air in different groups. R2^*^ **(A)** and R2 **(B)** values during room air are similar among different groups. R2' **(C)** values were greater in moderate (*P* = 0.032) and severe (*P* = 0.022) AKI groups than in control groups. ^*^P < 0.05; ns, not significant.

**Figure 5:**
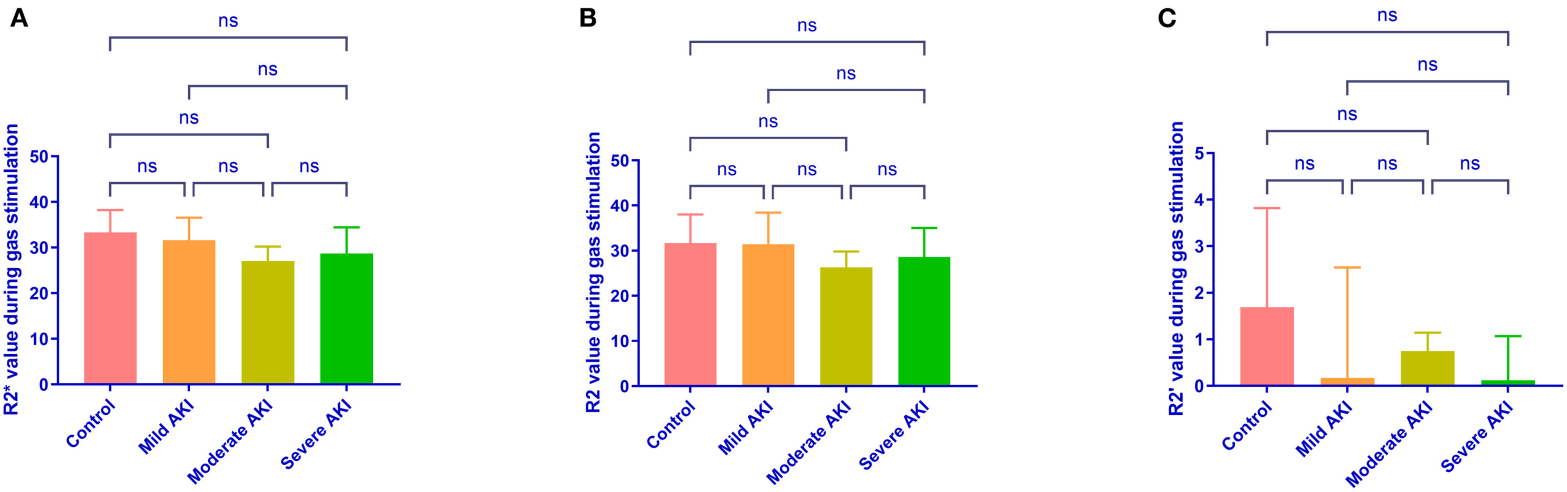
R2^*^, R2, and R2' values during carbogen in different groups. Differences are not identified in R2^*^ **(A)**, R2 **(B)** and R2' **(C)** values during carbogen among different groups. ns, not significant.

**Figure 6:**
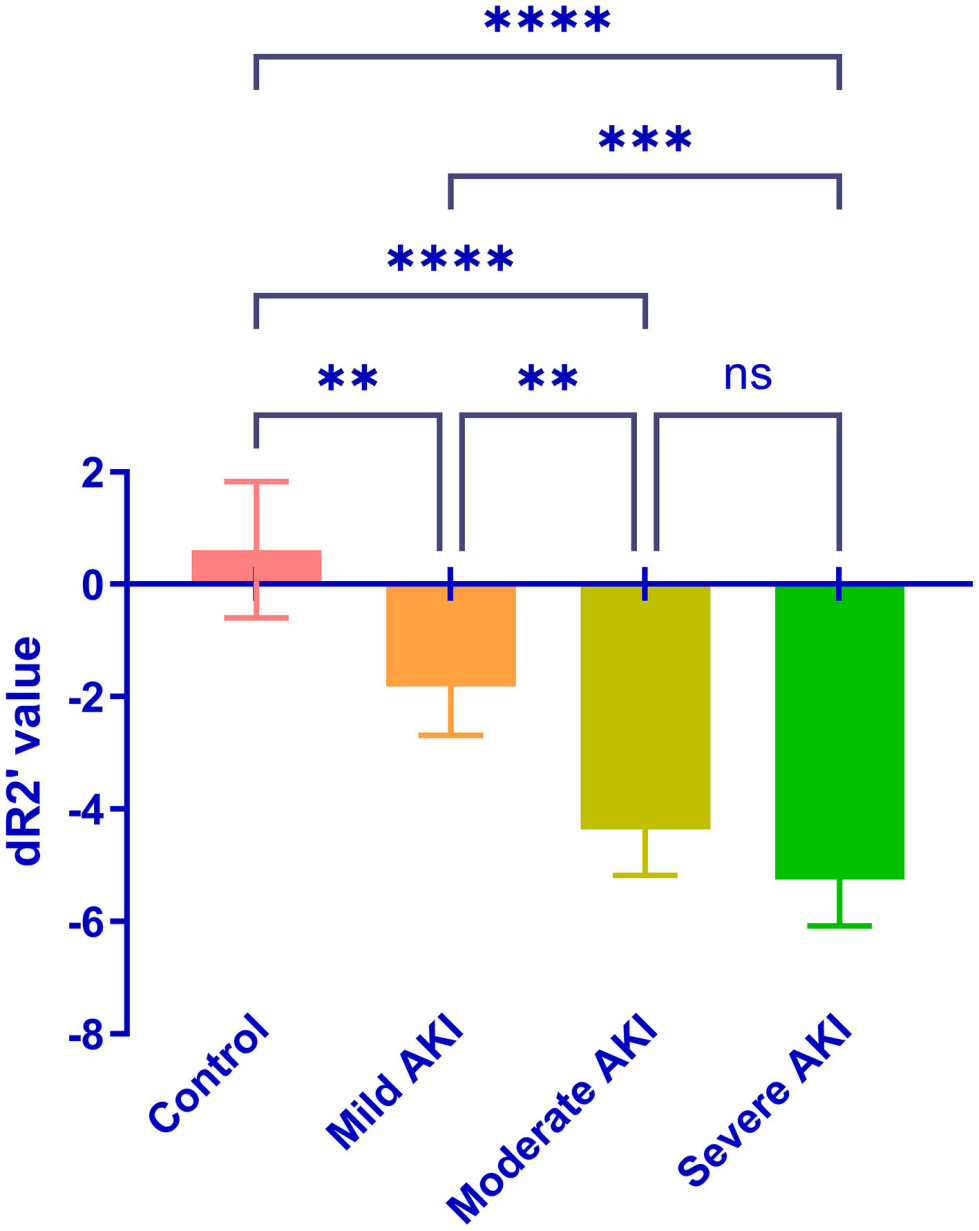
Comparison of the dR2' in the control and AKI groups. Significant differences were found by one-way ANOVA among groups (*P* < 0.0001). ^**^P < 0.01; ^***^P < 0.001; ^****^P < 0.0001; ns, not significant.

During room air, the values of R2^*^and R2 were similar among groups (*P* = 0.513 and 0.674, Figure 4). The values of R2' were different (*P* = 0.008). The R2' values between control and moderate/severe AKI groups differed significantly (control vs. moderate: *P* = 0.032, control vs. severe: *P* = 0.022), while the other comparisons' differences were not significant. During gas stimulation, the R2^*^, R2, and R2' values were all similar among different groups (*P* = 0.200, *P* = 0.800 and *P* = 0.442, respectively, Figure 5). The values of dR2' were 0.617 ± 1.216 (control), −1.818 ± 0.870 (mild), −4.365 ± 0.812 (moderate) and −5.251 ± 0.826 (severe). The values of dR2' were different among groups (*P* < 0.0001) and differences between every two groups were significant except the comparison of moderate vs. severe AKI groups (control vs. mild, *P* = 0.004; mild vs. moderate, *P* = 0.003; moderate vs. severe, *P* = 0.471; Figure 6). The values of ADC were 1.915 ± 0.052 in the control group, 1.735 ± 0.126 in the mild AKI group, 1.601 ± 0.055 in the moderate AKI group and 1.153 ± 0.075 in severe AKI group (*P* < 0.0001).

## Discussion

The present *in vivo* study demonstrates that it is feasible to use the combination of psMASE sequence with a modified HRI technique to diagnose and stage AKI in the very early phase. The values of dR2' were different between control and AKI groups and between AKI groups of different severities. R2^*^, R2, and R2' during room air and carbogen could not effectively distinguish different AKI severities. Compared with conventional R2^*^, R2, and R2', dR2' provided better diagnostic sensitivity.

Although R2^*^ can reflect oxygen content quantitatively, it is the sum effect of two components: R2, and R2'. R2 is sensitive to tissue water content, while R2' is sensitive to hemoglobin (11). Thus, it is more accurate to evaluate renal oxygenation by separating R2' (14). In this study, R2' is also better in distinguishing AKI than R2^*^ during room air. However, the signal intensity of BOLD MRI can be influenced by various factors, such as oxygen metabolism, blood flow and hematocrit (15). Therefore, it is defective to use the absolute value of R2' to evaluate the oxygen content. HRI technique can be used to monitor changes in renal perfusion and hemodynamics. Boss et al. reported a mild increase of 3–4% in T2^*^ signal intensity in bilateral kidneys in healthy volunteers during carbogen breathing (16). Milman et al. found a significant increase of 50% in T2^*^ during carbogen breathing (13). Variation of R2' during room air and carbogen was acquired in the present study by combining the psMASE sequence with a modified HRI technique. Differences of dR2' were found when absolute R2' values were still similar in different severities of AKI, which indicates the advantage of dR2' in sensitivity. Similar with our previous reports (8), the ADC values were also different in control and AKI groups and could not distinguish between different AKI stages effectively in the present study.

The changes of R2^*^ and R2' during the gas challenge are related to the hemodynamic mechanism of renal perfusion. In healthy kidneys, carbogen breathing will lead to the dilation of renal arteries, which increases the amount of oxygen transported to the kidney and decrease the proportion of deoxyhemoglobin. In AKI models, the increase of T2^*^ was significantly attenuated, corresponding with the decline in renal arterial blood flow velocity confirmed by Doppler US (13). The present study showed the dR2' value was higher in absolute values in more severe AKI groups. The results could be explained by better compensation capability in more healthy animals and different methods of establishing AKI models.

High temporal resolution is essential for the signal-to-noise ratio of HRI by improving the effectiveness of detecting signal changes and avoiding the accumulation of physical noise (17). Current methods of obtaining R2^*^ and R2 simultaneously have flaws, including low temporal resolution or significant estimation error. The applications of standard line-by-line Cartesian k-space acquisition approach of the gradient echo sampling of the free induction decay and echo (GESFIDE) and gradient echo sampling of the spin-echo (GESSE) sequences are restricted in fast dynamic real-time imaging (18, 19). This study implemented a gradient fast switching acquisition method to achieve quick filling and improve the imaging speed and temporal resolution. The spin- and gradient-echo (SAGE) sequence can also be used for fast imaging, but perfect matching of the slice profiles during multi-slice acquisitions is inaccessible (20). Signal acquisition was performed in the spin-echo formation and attenuation in the psMASE sequence in the present study, which avoided slice profile mismatch between images before and after 180° refocusing pulse caused by SAGE sequence and led to the more accurate acquisition.

Several new AKI biomarkers, including NGAL, KIM-1 and IL-18 have been discovered and validated to improve early diagnosis and degree of severity (4). Urinary NGAL has been found to be a powerful early marker of AKI in children with receiver operating characteristics level > 0.99 (21). KIM-1 and IL-18 are also reported to be associated with AKI in the early phase (22, 23). However, the failue of validating the diagnostic values of these biomarkers in large multicenter trials made the clinical use of these biomarkers unclear (4, 24). The use of dR2' in early diagnosis of AKI has not been validated in human subjects until now. The accuracy and price in patients of different pathophysiological conditions are unknown and there is still a long journey (4).

Limitations are present in this study. First, AKI can be caused by multiple pathological factors, and ischemic animal models cannot reflect all AKI patients' actual conditions. Patients with AKI are needed to validate the feasibility of this method in further studies. Second, although differences of dR2' were found among groups in our study, more evidence is needed to prove that the parameter can be used to diagnose and stage AKI in clinical scenarios and the sensitivity and specificity are unknown.

## Conclusion

In conclusion, a combination of the psMASE sequence and HRI imaging was performed in animal AKI models. This modified imaging approach is feasible in diagnosing and staging AKI in the early phase and can be a potential non-invasive imaging biomarker for AKI in clinical settings.

## Data Availability Statement

The raw data supporting the conclusions of this article will be made available by the authors, without undue reservation.

## Ethics Statement

The animal study was reviewed and approved by Peking University First Hospital Animal Ethics Committee.

## Author Contributions

BZ and ZY contributed to the implementation. WG, CW, and HK contributed to data analysis. JZ and MY contributed to designing and supervising. All the authors contributed to writing and revising the manuscript.

## Funding

This work was supported by the National Natural Science Foundation of China under grant [Grant Number: 81571666], Interdisciplinary Clinical Research Project of Peking University First Hospital [Grant Number: 2018CR33], Scientific Research Seed Fund of Peking University First Hospital [Grant Number: 2018SF023], Youth Clinical Research Project of Peking University First Hospital [Grant Number: 2018CR16].

## Conflict of Interest

The authors declare that the research was conducted in the absence of any commercial or financial relationships that could be construed as a potential conflict of interest.

## Publisher's Note

All claims expressed in this article are solely those of the authors and do not necessarily represent those of their affiliated organizations, or those of the publisher, the editors and the reviewers. Any product that may be evaluated in this article, or claim that may be made by its manufacturer, is not guaranteed or endorsed by the publisher.
